# A Critical Analysis of the Evidence for the SARS-CoV-2 Origin Hypotheses

**DOI:** 10.1128/msphere.00119-23

**Published:** 2023-03-28

**Authors:** James C. Alwine, Arturo Casadevall, Lynn W. Enquist, Felicia D. Goodrum, Michael J. Imperiale

**Affiliations:** a Department of Cancer Biology, University of Pennsylvania, Philadelphia, Pennsylvania, USA; b Department of Immunobiology, University of Arizona, Tucson, Arizona, USA; c Bloomberg School of Public Health, Johns Hopkins University, Baltimore, Maryland, USA; d Department of Molecular Biology, Princeton University, Princeton, New Jersey, USA; e BIO5 Institute, University of Arizona, Tucson, Arizona, USA; f Department of Microbiology and Immunology, University of Michigan, Ann Arbor, Michigan, USA

**Keywords:** COVID-19, SARS-CoV-2, zoonosis, coronavirus, lab leak, origin

## Abstract

When humans experience a new, devastating viral infection such as severe acute respiratory syndrome coronavirus 2 (SARS-CoV-2), significant challenges arise. How should individuals as well as societies respond to the situation? One of the primary questions concerns the origin of the SARS-CoV-2 virus that infected and was transmitted efficiently among humans, resulting in a pandemic. At first glance, the question appears straightforward to answer. However, the origin of SARS-CoV-2 has been the topic of substantial debate primarily because we do not have access to some relevant data. At least two major hypotheses have been suggested: a natural origin through zoonosis followed by sustained human-to-human spread or the introduction of a natural virus into humans from a laboratory source. Here, we summarize the scientific evidence that informs this debate to provide our fellow scientists and the public with the tools to join the discussion in a constructive and informed manner. Our goal is to dissect the evidence to make it more accessible to those interested in this important problem. The engagement of a broad representation of scientists is critical to ensure that the public and policy-makers can draw on relevant expertise in navigating this controversy.

## EDITORIAL

The Merriam-Webster online dictionary defines a hypothesis as a tentative assumption made to draw out and test its logical or empirical consequences. Hypotheses are part of the scientific method and are formulated to be tested by well-designed, controlled experiments that will support or refute them. The most powerful experiment is one that has the potential to eliminate a hypothesis or to reveal a deep flaw in it. Many experiments lack this potential and at best can only provide data that are consistent with a given hypothesis. While coincidences, correlations, or temporal sequences of events can be useful for formulating a hypothesis, they leave room for alternative explanations and are not conclusive. In the case of the origin of severe acute respiratory syndrome coronavirus 2 (SARS-CoV-2), the primary candidate hypotheses are not readily falsifiable with the currently available information; thus, we must acknowledge that a definitive determination of the origin is unlikely.

Two major hypotheses regarding the origin of SARS-CoV-2 have been debated: a direct zoonotic origin and the introduction of the virus into humans from a laboratory source (1–5). The zoonosis hypothesis states that the virus originated naturally from an animal-to-human spillover event followed by sustained human-to-human transmission. This scenario has happened repeatedly over time, causing epidemics and pandemics, including influenza, SARS, Middle East respiratory syndrome (MERS), Ebola, HIV, as well as bacterial infections like bubonic plague. Viral emergence events through zoonoses often occur when host species alter their geographical range in response to environmental changes, habitat destruction, or urbanization. Hot spots for emergence in the human population exist in regions with high human population densities and frequent contact between humans and wildlife or agricultural species that harbor zoonotic pathogens. Only a small proportion of viruses that spill over into humans result in disease, and even fewer result in an outbreak with sustained human-to-human transmission. Most initial transmission events evade detection because they do not cause disease, do not come to medical attention, or are misdiagnosed. The lab leak hypothesis postulates that researchers constructed or simply cultured SARS-CoV-2 during research on bat-origin coronaviruses (CoVs) and that an accidental exposure to this lab-derived virus started the pandemic.

It is worthwhile to consider what type of evidence would definitively rule in or rule out either the zoonotic or lab leak hypothesis. Scientific evidence alone is likely to be insufficient to provide a definitive answer (1). In fact, the types of evidence needed for ruling in and out the two hypotheses are quite different. Establishing the lab leak hypothesis would require evidence that the Wuhan Institute of Virology (WIV) was working on a CoV very closely related to the original Wuhan strain, and such evidence would have to come from laboratory records. Had the WIV been working on such a virus, evidence of a laboratory accident and/or that some of the initial cases had come from individuals at the WIV would strongly support the possibility of a lab leak. While the Chinese government has denied that such work was being done by the WIV, transparency is lacking (2). Unless such evidence is forthcoming, the laboratory leak hypothesis cannot be confirmed. Conclusively establishing the zoonotic hypothesis requires finding evidence of the original animal-to-human infection event(s). This is difficult because these events likely occurred unnoticed, and there may be no record of their eventuality. However, the recovery of a CoV that is closely related to SARS-CoV-2 from bats and/or intermediate hosts would support the hypothesis that the coronavirus disease 2019 (COVID-19) pandemic was initiated by a zoonotic event. As discussed below, however, the needed exploration of multiple species for CoV prevalence and diversity will take time to achieve.

In addition to requiring very different sources of data, we must realize that the levels of certainty required to definitively prove either the lab leak or the zoonotic hypothesis differ significantly. Specifically, it should be possible to rule in or out the lab leak theory with information from the WIV, if such information exists. In contrast, unequivocally ruling in or out the zoonotic explanation is not possible since the evidence will always depend on certain probabilities, and certainty is impossible without knowledge of the initial events.

In this light, the only way to assess the viability of the hypotheses is to examine the best available evidence. It is important that scientists, the public, and public figures follow the facts and limit speculation that can become fodder for misinformation and conspiracy theories. In most instances, this is how science proceeds, following and building upon the best available evidence but always maintaining an open mind since all scientific knowledge is provisional, and the next experiment might change our opinions dramatically. In applying the scientific method, we ask a question, form a hypothesis, make a prediction, test the prediction, and then use the results to make a new hypothesis. The iterative nature of scientific investigation requires constantly revisiting previous conclusions as new data are collected. Scientific findings based on this philosophy are our best tool for directing our resources for effective responses to the many challenges that face our society. The scientific method has been broadly applied by scientists the world over to understand the origin of SARS-CoV-2.

To explore the origin question, we pose several relevant hypotheses below and critically consider the scientific data to support or refute them. Other evidence may emerge from ongoing investigations carried out by the scientific community, intelligence community, and government agencies that will hopefully shed further light on the SARS-CoV-2 origin question. It is important that as more evidence emerges, we revisit and reshape conclusions to reflect new data.

## HYPOTHESIS 1: SARS-CoV-2 AROSE FROM A LABORATORY-ADAPTED CoV

### Background.

The genomic sequence of SARS-CoV-2 offers a means of testing the laboratory-origin hypothesis. Namely, if the virus had a laboratory origin, we would expect the viral genome to carry signatures resulting from the propagation of the biological isolate in the lab. This expectation is based on the reasoning that if the isolate were present in the lab but not propagated, the potential for laboratory-acquired infection would be extremely low due to the limited handling of the sample and likely low viral titers.

### Evidence A: loss of the furin cleavage site in the spike protein during cell culture adaptation of initial isolates of SARS-CoV-2.

Over the past 3 years, hundreds of research groups around the world have cultured human isolates of SARS-CoV-2 in cell lines. This collective experience has revealed a highly reproducible consequence of viral propagation in culture: the deletion of the portion of the spike gene encoding the furin cleavage site (3). If SARS-CoV-2 had a laboratory origin, it would have been amplified in a laboratory through a process of serial passage typically needed to recover high-titer stocks from environmental samples. In this process, the deletion of the furin cleavage site is expected, offering a signature of laboratory handling. However, early isolates of SARS-CoV-2 show the furin cleavage site to be intact, arguing against introduction into humans after laboratory cell culture.

### Evidence B: initial SARS-CoV-2 isolates replicate poorly in traditional laboratory models.

Traditional laboratory experiments would likely have included the adaptation of a virus in common laboratory animal models such as mice to study it with ease. Thus, a laboratory-derived virus released into the population would reasonably be expected to carry these adaptive markers in their genomes. However, early isolates of SARS-CoV-2 did not carry mutations that confer adaptation to common animal models, such as mutations in the receptor binding domain of spike that improve viral replication in mice (4, 5).

## HYPOTHESIS 2: SARS-CoV-2 IS A LABORATORY-CONSTRUCTED VIRUS

### Background.

CoV can be modified or generated from sequence information using molecular biology techniques (6). The existence of this technology has led some to suggest that SARS-CoV-2 not only may have been propagated in a lab but also may have been generated *de novo* in a lab. In this context, the furin cleavage site has been highlighted as a feature that may have been designed to increase virulence.

### Evidence A: lack of any evidence of deliberate genetic engineering.

Genetic engineering involves the use of recombinant DNA techniques to modify the virus. Such techniques often leave telltale evidence in the forms of novel restriction sites, differences in DNA base content, differences in gene organization from natural isolates, selection markers, and foreign nonviral sequences. While various papers have suggested that SARS-CoV-2 carries hallmarks of recombinant DNA technologies, this theory has been thoroughly refuted (7), including by the U.S. intelligence community.

### Evidence B: the pathogenesis of SARS-CoV-2 may be dependent on the cleavage site loop length and not only the presence of the furin cleavage site.

The furin cleavage site is an important determinant of virulence (4). A furin cleavage site is absent from the closest known relatives of SARS-CoV-2; however, these closest relatives are, in fact, distantly related. Furin cleavage sites are commonplace in other CoV spike proteins, including those of endemic human betacoronaviruses, and were first identified in the 1980s within a mouse coronavirus (8). Prior to the COVID-19 pandemic, it was commonly thought that the presence of the furin cleavage site was directly linked to the pathogenesis of CoV. However, a recent examination of SARS-CoV-2 has revealed a somewhat complex relationship between the presence of a furin cleavage site and viral pathogenicity. In fact, the context of the cleavage site defines the extent to which this site drives virulence. Specifically, the amino acids N terminal to the cleavage site, which comprise the cleavage loop, are important: both the loop’s length and the presence of a glycosylation site within it define the contribution of the furin cleavage site to virulence (9). Since these nuances were not understood prior to 2020, deliberate engineering of the SARS-CoV-2 cleavage site to promote pathogenesis without this critical information is improbable.

### Evidence C: loss of the furin cleavage site through cell culture propagation of the virus.

As mentioned above in the evidence for hypothesis 1, the propagation of the virus in standard cell culture results in a deletion of the furin cleavage site. Constructing a virus in a laboratory would necessarily require that it be grown in a lab, where the site would likely be deleted, yet the subsequently lab-grown virus population must retain the furin cleavage site. This contradictory situation, as well as the fact that SARS-CoV-2 isolates retain a furin cleavage site, suggests that the virus had not been cultured in a lab at the time of emergence.

## HYPOTHESIS 3: SARS-CoV-2 IS A BAT ZOONOSIS INTRODUCED INTO THE HUMAN POPULATION

### Background.

It is widely accepted that bat species contain a large diversity of coronaviruses. Since the SARS-CoV outbreak in 2003, we furthermore know that bats act as a reservoir for CoVs that pose the potential for spillover. For this reason, bats have been subject to large-scale surveillance since 2003. In addition to CoVs, many human viruses have their origins in nonhuman hosts. Influenza A viruses are a pertinent example owing to the recurrent nature of influenza pandemics. Our most recent influenza pandemic, in 2009, was caused by a virus circulating in pigs. While pigs were known to harbor influenza viruses with zoonotic potential, and the 2009 strain showed similarities to swine strains, a genetically similar precursor virus was not identified in swine until 2016 (10).

### Evidence A: a bat origin is highly plausible given the precedent of SARS-CoV and the diversity of CoVs in global bat populations.

The source of the SARS-CoV outbreak of 2002 to 2004 is particularly relevant to consider given the similarities between this virus and SARS-CoV-2. In the years following the SARS-CoV outbreak, extensive molecular epidemiology and wildlife surveillance efforts determined its proximal source to be civet cats sold within live-animal markets in Guangdong, China (11). The civet cats, in turn, were likely to have been infected by bats (12). Although an intermediate animal host for SARS-CoV-2 has not been identified, a similar path of emergence from bats to wild animals to humans, facilitated by live-animal markets, is feasible. Viruses related to SARS-CoV-2 have been documented in multiple locations in Southeast Asia, primarily in bats (13–18). These data show that bats harbor a diverse gene pool of CoVs and are likely to have sustained circulation of the precursor lineage.

### Evidence B: bat CoVs identified to date are not closely related to SARS-CoV-2.

Phylogenetic analyses reveal a significant evolutionary gap between SARS-CoV-2 and the most closely related viruses sampled to date from bats. Bat CoVs were under study at the Wuhan Institute of Virology (WIV) in the period preceding the pandemic as part of an ongoing important surveillance effort. The most closely related virus known to be in the WIV collection, RaTG13, differs from early SARS-CoV-2 isolates by >1,000 nucleotides, a genetic distance of ~4% (19). Given this dissimilarity, RaTG13 is clearly not a proximal ancestor of SARS-CoV-2. However, owing to a lack of transparency, independent verification of the viruses present in the laboratories of the WIV is lacking. Further surveillance in bats since 2020 has identified additional CoVs that are related to SARS-CoV-2 (13–18, 20). Because surveillance coverage is far from complete, however, large gaps remain in our knowledge that preclude the identification of the viral lineage that seeded the pandemic or the intermediate host: the host niche harboring the direct precursors of SARS-CoV-2 has not yet been adequately sampled. It is important to recognize that interpreting this failure to identify a feral source of SARS-CoV-2 as evidence supporting a laboratory origin constitutes the logical error of using the absence of evidence as evidence for an absence. We note that, as for the 2009 influenza pandemic, it took many years to identify the feral sources of SARS-CoV and HIV after their initial isolation from humans (19, 21).

### Evidence C: high rates of recombination of CoVs occur in nature.

Recombination within the *Sarbecovirus* subgenus to which SARS-CoV-2 belongs is exceedingly common (18, 22, 23). Recombination naturally produces chimeric genomes, making lineage tracing to ancestors more complex, but also increases the natural diversity of CoVs. Thus, signatures of recombination in the SARS-CoV-2 genome do not point specifically to either a zoonotic or a laboratory origin.

### Evidence D: numerous animal reservoirs for CoVs and SARS-CoV-2 are known to exist in nature.

During the COVID-19 pandemic, many animal hosts at the human-animal interface were identified to be susceptible to the virus, such as cats and deer (24, 25). However, an analysis of animals at the Wuhan market at the time of the outbreak did not identify a single species as the intermediate host (26). SARS-CoV-2 sequences were detected in environmental samples obtained from cages and stalls where live animals susceptible to SARS-CoV infection had been kept, but those animals were no longer present (26). The broad tropism of CoVs in nature has led to speculation of pangolins and snakes as the intermediate hosts. The lack of surveillance data prior to the outbreak limits the ability to identify a possible intermediate host.

### Evidence E: serological evidence for human exposure to bat viruses.

Antibodies to bat coronavirus have been detected in humans living near caves in China, providing immunological evidence for the transmission of CoVs from bats to humans directly or through intermediate hosts (27). Recently, a novel rhabdovirus was isolated from bats, and virus-specific antibodies were detected in people who lived near the collection site, providing more evidence that bats can be a source of viruses that infect humans (27).

## HYPOTHESIS 4: THE SARS-CoV-2 ORIGIN IS SUGGESTED BY EARLY CASES IN THE COVID-19 OUTBREAK

### Background.

The COVID-19 pandemic began in central Wuhan, a densely populated megacity of over 11 million people. At least four markets in Wuhan traded live wild animals in the months preceding the pandemic (2), an activity that brings large numbers of people into proximity with a diversity of wild animals.

### Evidence A: known cases cluster at or near the Huanan Seafood Wholesale Market.

Among the first 174 documented cases of SARS-CoV-2 infection in December 2019, approximately one-half had a recent association with a market, and approximately one-quarter had an association with the Huanan Seafood Wholesale Market in particular (2). None were scientists working on bat CoVs. Importantly, this set of early cases is almost certainly incomplete since many SARS-CoV-2 infections are asymptomatic, and mild cases are difficult to distinguish clinically from other respiratory infections. However, both in terms of a direct association and in considering geographical proximity, the early stages of the outbreak showed a clear link to the Huanan market (26).

### Evidence B: emergence of two lineages of SARS-CoV-2.

Early cases in Wuhan involved two lineages, distinguishable by the viral genome sequence, suggesting that there were multiple events of spillover into humans. This situation is more likely to have resulted from zoonosis than a lab accident as lab accidents are relatively rare, and both lineages of SARS-CoV-2 were found in sequences from the Hunan market (28). Additionally, the extreme transmission heterogeneity of SARS-CoV-2 is important to consider. Epidemiological analysis of the pandemic indicates that 80% of infections do not lead to onward transmission (29). This heterogeneity suggests that many spillover events, rather than a single introduction through an infected lab worker, were likely needed to initiate sustained spread.

### Evidence C: lack of epidemiological information from the WIV.

Because data are not available, it is unclear how comprehensively Wuhan-based scientists working on CoVs were tested. While it has been reported that three scientists from the WIV had a respiratory illness in the fall of 2019 (30), they were not tested for any specific pathogen, and therefore, the causative agent of their illness cannot be determined. Human respiratory illnesses are common in the general population and can be caused by several viruses such as influenza virus, parainfluenza virus, and respiratory syncytial virus (RSV) as well as some bacteria and fungi. For many respiratory illnesses, the infectious etiology is never known.

## CONCLUSIONS DRAWN FROM EXISTING EVIDENCE

Scientific conclusions are based on likelihood given the scientific data, and conclusions can change as new data are obtained. Based on the scientific data collected in the last 3 years by virologists worldwide, hypotheses 1 and 2 are unlikely. Hypotheses 3 and 4 cannot be ruled out by existing evidence. Since hypotheses 1 and 2 support the lab leak theory and hypotheses 3 and 4 are consistent with a zoonotic origin, the lab leak- and zoonotic-origin explanations are not equally probable, and the available evidence favors the latter. Further insight into CoVs in animals at the animal-human interface requires additional surveillance of circulating virus sequences from animals. There is ample precedent for the seeding of pandemics and more geographically limited outbreaks from nonhuman species. Common-cold CoVs, SARS-CoV, Ebola virus, HIV, influenza A virus, mpox virus, and others all have zoonotic origins (31–33). SARS-CoV-2 is the ninth documented coronavirus to enter the human population. The best existing scientific evidence supports a direct zoonotic origin. As new evidence continues to emerge from scientific studies or other investigations, our understanding of the origin of SARS-CoV-2 will continue to evolve. Nevertheless, it is possible that its origin may never be known with certainty.

## CONCLUDING REMARKS

The COVID-19 pandemic brought tremendous suffering to humanity. It is reasonable to want to hold an individual(s) accountable, and it may be unsatisfying to attribute such calamities to natural causes. We understand the anger that people feel because of losing or being separated from loved ones, homeschooling their children, and having their lives upended. We have lived that experience as well. Science was our best ally during the pandemic, working to understand virus replication, spread, and disease to produce life-saving vaccines, antivirals, rapid tests, and treatments. Science is also a key ally in addressing origin hypotheses. Given the currently available evidence, the two plausible possibilities for the origin of SARS-CoV-2 are not equally likely. Continuing to frame them as such does us all a disservice, dilutes our efforts, and misdirects resources—only serving to weaken our ability to respond to future pandemics.
